# Clinical Characteristics and Outcomes of Multiple Sclerosis and Neuromyelitis Optica Spectrum Disorder With Brainstem Lesions as Heralding Prodrome

**DOI:** 10.3389/fneur.2022.836337

**Published:** 2022-05-09

**Authors:** Qiling Ji, Huiqing Dong, Hangil Lee, Zheng Liu, Yanna Tong, Kenneth Elkin, Yazeed Haddad, Xiaokun Geng, Yuchuan Ding

**Affiliations:** ^1^Department of Neurology, Beijing Luhe Hospital, Capital Medical University, Beijing, China; ^2^Department of Neurology, Xuanwu Hospital, Capital Medical University, Beijing, China; ^3^Department of Neurosurgery, Wayne State University School of Medicine, Detroit, MI, United States

**Keywords:** brainstem, multiple sclerosis, neuromyelitis optica spectrum disease, demyelination, image characterization

## Abstract

**Objective:**

The present study sought to differentiate multiple sclerosis and neuromyelitis optica spectrum disorder patients at their first attack by describing and distinguishing their clinical features, radiographic characteristics, and immunologic characteristics of serum and cerebrospinal fluid.

**Methods:**

We retrospectively studied 58 patients with multiple sclerosis (MS) and 52 patients with neuromyelitis optica spectrum disorder (NMOSD) by referencing brainstem lesions as the prodromal events. Their demographics and presentation at the time of the first attack was evaluated including their gender, age, clinical features of the first attack, the expanded disability status scale (EDSS), brainstem lesion(s) by head MRI, and immunological indices of serum and cerebrospinal fluid.

**Results:**

The NMOSD group had more female patients (4.8 vs. 1.9, *p* < 0.05), and was older than the MS group (37.81 ± 16.60 vs. 27.57 ± 11.17, *p* <0.001). NMOSD patients also had a significantly higher association with autoimmune diseases or positive autoimmune antibodies (*p* < 0.01). There was no significant difference in the EDSS scores between the two groups (*p* = 0.420). Central hiccups, vomiting, and pyramidal tract signs were more common in the NMOSD group than the MS group (*p* < 0.001, *p* < 0.001, *p* < 0.01), while eye movement abnormalities were more common with MS (*p* < 0.01). There were no significant differences in other clinical manifestations such as vertigo, diplopia, limb weakness, numbness, and eating difficulty. MS patients were more likely to have midbrain and pons imaging lesions (*p* < 0.001, *p* < 0.001), while NMOSD patients had more lesions in the medulla oblongata (*p* < 0.001). The lesions in the MS group were mostly located in the periphery, while those in the NMOSD group were centrally located (*p* < 0.001, *p* < 0.001). Patchy lesions were more common in MS patients (*p* < 0.001), while large lesions were more common in the NMOSD group (*p* < 0.001). Finally, serum AQP4 Ab was found only in the NMOSD group (*p* < 0.001).

**Conclusion:**

Patients with MS and NMOSD have differentiating clinical manifestations at the time of their first brainstem lesions which include central hiccups, vomiting, pyramidal tract signs, and abnormal eye movements. Additionally, distinct imaging manifestations such as lesion location(s) and morphology may also aid in the development of pathognomonic criteria leading to timely initial diagnosis of MS and NMOSD.

## Introduction

Multiple sclerosis (MS) is a common demyelinating disease in the central nervous system (CNS) with a relapsing and remitting clinical course. Its incidence rate and morbidity are the highest among CNS demyelinating disorders. Previously, neuromyelitis optica (NMO) was considered as a subtype of MS that mainly affected the optic nerves and spinal cord. However, recent clinical, imaging, and immunologic data indicated that NMO is a distinct disease with a unique pathogenesis (1, 2). The discovery of anti-aquaporin-4 (AQP4) antibody (Ab) was essential for the evolution of NMO diagnostic criteria, leading to its reclassification as a member of a larger group of disorders called neuromyelitis optica spectrum disorders (NMOSD) (3).

Most lesions in MS patients involve the spinal cord, paraventricular region, cerebral cortex and subcortex, infratentorial region, and optic nerves. Although there are some similarities in NMOSD patients as they also have lesions in the spinal cord and the optic nerves, they are distinguished by involvement of area postrema, diencephalon, and posterior cortex. Both can involve the brainstem and exhibit corresponding clinical manifestations. Although they have some distinguishing clinical and imaging manifestations, they have significant overlap, making their differentiation challenging (1, 4).

As the pathogeneses of MS and NMOSD differ widely, their prognoses and treatment approaches are also consequently unique; hence, it is crucial to distinguish them at time of onset to optimize their care (3, 5–11). However, it is often difficult to distinguish between MS and NMOSD at their initial presentation with mainly brainstem involvement due to their overlapping clinical and radiographic manifestations. Although immunological indices are helpful for their differentiation, obtaining them at the time of disease onset is often unfeasible. Clinical symptoms and imaging are typically the most readily available information to clinicians, so it would be particularly helpful to be able to distinguish the two by those information alone.

Therefore, we compared and analyzed the clinical features and imaging manifestations of MS and NMOSD in patients when they first presented with brainstem lesion symptoms, aiming to delineate their unique characteristics to distinguish the two diseases at symptom onset. This work paves the way to expedite proper treatment administration and improve patient prognosis in the future.

## Subjects and Methods

### Subjects

The present study included 58 patients diagnosed with multiple sclerosis(MS)and 52 patients with neuromyelitis optica spectrum disorders (NMOSD) who were admitted to Xuanwu Hospital Capital Medical University between January 2013 and June 2019. Patients with MS met the clinical diagnostic criteria of McDonald 2010 (12) and patients with NMOSD met the diagnostic criteria set by the International Panel for NMO Diagnosis (IPND) in 2015 (1). All patients presented initially with brainstem lesions and underwent MRI imaging, cerebrospinal fluid (CSF) testing, and serological testing within 1 month of symptom onset. MRI imaging included T1 weighted, T2 weighted, and T2 flair, with or without gadolinium contrast enhancement. Cerebrospinal fluid and serological testing included the cell-based transfer immunofluorescence assay (CBA) for AQP4 Ab detection and isoelectric focusing electrophoresis for cerebrospinal fluid specific oligoclonal band (SOB) detection. Patients with non-idiopathic demyelinating disease were excluded from the study.

### Methods

This study is a retrospective analysis. Patients that met the respective diagnostic criteria with appropriate clinical data were included. A questionnaire was completed for each patient, which included the patient's gender, age, follow-up time, relapse times, presence of an autoimmune disease or autoimmune antibodies at the time of onset, EDSS score at onset, clinical signs and symptoms, and MRI images. Symptoms such as vertigo, pupillary miosis, nystagmus, diplopia, medial longitudinal fasciculus (MLF) syndrome, numbness of limbs, numbness of mouth and face, headache, dysphagia (coughing after eating), dysarthria (slurred speech), weakness of limbs, facial paralysis, central hiccups, central vomiting, pyramidal tract signs, sensory disturbance, ataxia, ophthalmoplegia (abnormal eye movements), and bulbar paralysis were regarded as manifestations of brainstem symptoms. On the MRI images, the lesion location, shape, size, and enhancement were noted. Immunological indices from serum and CSF were collected including the presence of cerebrospinal fluid specific oligoclonal bands (SOB), serum and cerebrospinal fluid AQP4 antibody, and myelin basic protein (MBP).

### Statistical Analyses

The SPSS 17.0 software was utilized for statistical analyses. Data with normal distribution were expressed as mean ± standard deviation and pairwise comparisons between groups were performed with the LSD t-test. The data of non-normal distribution was expressed as median with interquartile intervals. The Bonferroni correction was performed for the pairwise comparisons between groups. The Chi square test was used for categorical data. *P* <0.05 was used as the standard of statistical significance in all cases.

## Results

### Patient Demographics

The NMOSD group had a statistically significant greater ratio of female patients as compared to the MS group, although there were no significant differences in follow-up times and episode frequencies. In the MS group, typical onset was from juvenile to middle-aged without any patients over 60 years old. NMOSD patients' onset were juvenile to elderly, but no patients over 65 years old were found; furthermore, NMOSD age of onset had a bimodal distribution with peaks at 16–30 years old and 61-65 years old (Table 1).

**Table 1:** General information of patients.

	**MS** **(*n* = 58)**	**NMOSD** **(*n* = 52)**	***P***
Age/(year)	27.57 ± 11.17	37.81 ± 16.60	<0.001*
Sex (male: female)	1:1.9	1:4.8	0.041*
Median follow-up time/ (month)	5.5 (2–108)	6 (2–80)	0.814
Median of episodes	2 (2–7)	2 (1–8)	0.614

* *P <0.05*.

### Clinical Manifestations

There were no statistically significant differences in the first EDSS score between the two groups. However, 15% of the NMOSD cohort was found to have an autoimmune disease or tested positive for an autoimmune antibody, while none from the MS group met these criteria (*p* = 0.006). There were several clinical symptoms with statistically significant differences between the two groups. The NMOSD group had more central hiccups (*p* < 0.001), central vomiting (*p* < 0.001), and pyramidal tract signs (*p* = 0.004), while the MS group had more MLF syndromes (*p* = 0.013) and ophthalmoplegias (*p* = 0.004) (Table 2).

**Table 2:** General information and clinical features of patients.

**Projects**	**MS** **(*n* = 58)**	**NMOSD** **(*n* = 52)**	***P***
With autoimmune disease or antibody, *n* (%)	0	8 (15.38)	0.006*
First EDSS score	2.0 (0–8.5)	2.0 (0–9.5)	0.420
Clinical manifestations, *n* (%)			
Vertigo	20 (34.48)	18 (34.62)	0.988
Pupillary miosis	0	0	
Nystagmus	15 (25.86)	14 (26.92)	0.900
Diplopia	26 (44.83)	14 (26.92)	0.051
MLF syndrome	18 (31.03)	6 (11.54)	0.013*
Paresthesia	18 (31.03)	10 (19.23)	0.156
Numbness of limbs	12 (20.69)	6 (11.54)	0.195
Numbness of mouth and face	14 (24.14)	6 (11.54)	0.087
Headache	4 (6.90)	0	0.156
Trigeminal neuralgia	1 (1.70)	0	1.000
Dysphagia (coughing after eating)	4 (6.90)	4 (7.69)	1.000
Dysarthria (slurred speech)	4 (6.90)	6 (11.54)	0.608
Weakness of limbs	10 (17.24)	14 (26.92)	0.220
Facial paralysis	2 (3.45)	0	0.524
Central hiccups	0	26 (50.00)	<0.001*
Central vomiting	4 (6.90)	34 (65.38)	<0.001*
Clinical signs, *n* (%)			
Pyramidal tract sign	10 (17.24)	22 (42.31)	0.004*
Sensory disturbance	18 (31.03)	10 (19.23)	0.156
Ataxia	5 (8.62)	0	0.088
Ophthalmoplegia (abnormal eye movements)	26 (44.83)	10 (19.23)	0.004*
Bulbar paralysis	4 (6.90)	4 (7.69)	1.000

* *P <0.05.*

*MLF syndrome: Medial Longitudinal Fasciculus syndrome.*

*Ophthalmoplegia (Abnormal eye movements): abnormal positioning of the eyeball(s) and/or gaze movements in any directions. This includes unilateral or bilateral nuclear, internuclear, and supranuclear ophthalmoplegia*.

### Radiographic Manifestations

**Figure 1:**
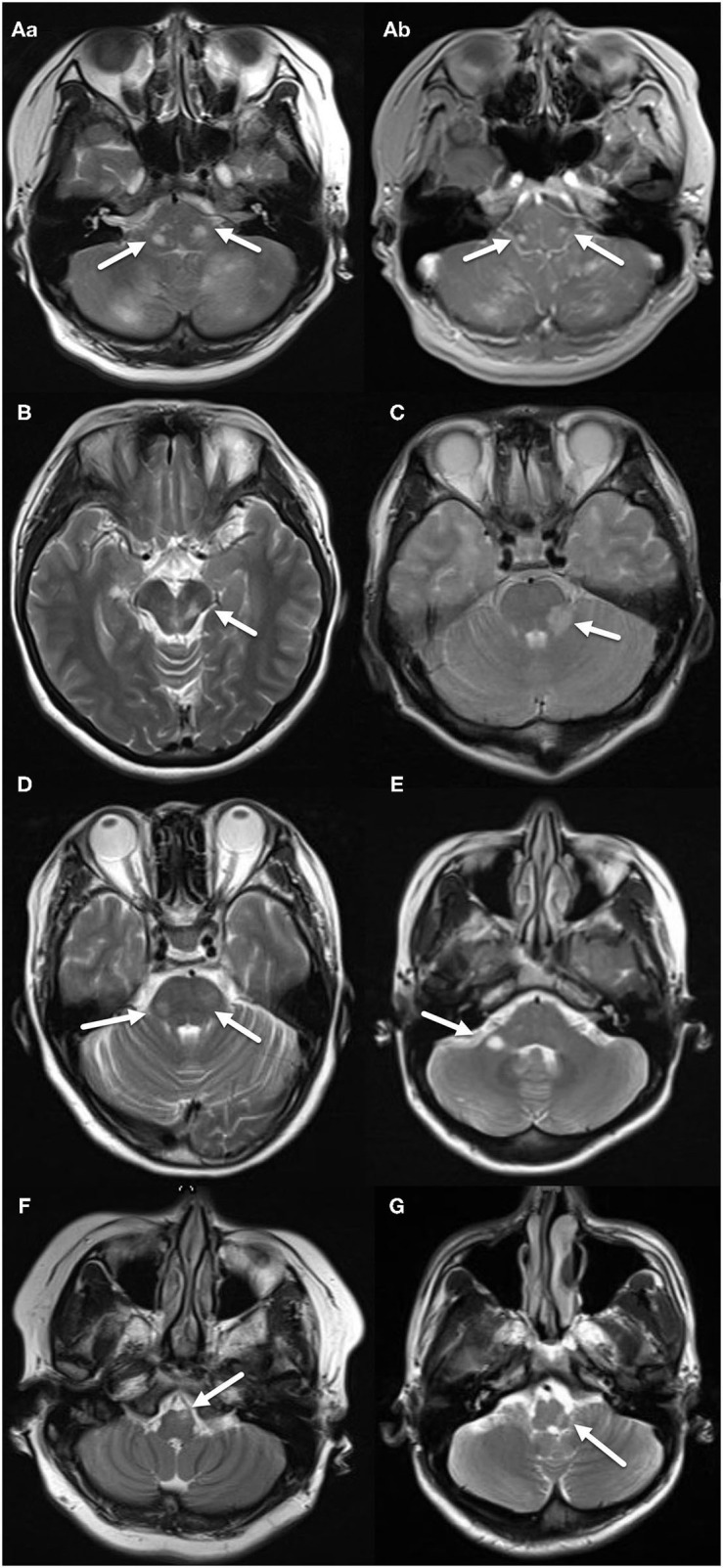
MRI features of the MS group. (**A**–a) Two patchy, abnormal signals located on both sides of pons, indicated by arrows. (**A**–b) Two nodular enhancing lesions located on both sides of pons, indicated by arrows. **(B)** Patchy, abnormal signal located in tegmentum of midbrain, indicated by an arrow. **(C)** Patchy, abnormal signal located in left brachium pontis, indicated by an arrow. **(D)** Two patchy, abnormal signals located on both sides of pons, indicated by arrows. **(E)** Ovoid, abnormal signal located in right brachium pontis, indicated by an arrow. **(F)** Patchy abnormal signal located in left ventral medulla oblongata, indicated by an arrow. **(G)** Patchy abnormal signal located in left dorsal medulla oblongata, indicated by an arrow.

**Figure 2:**
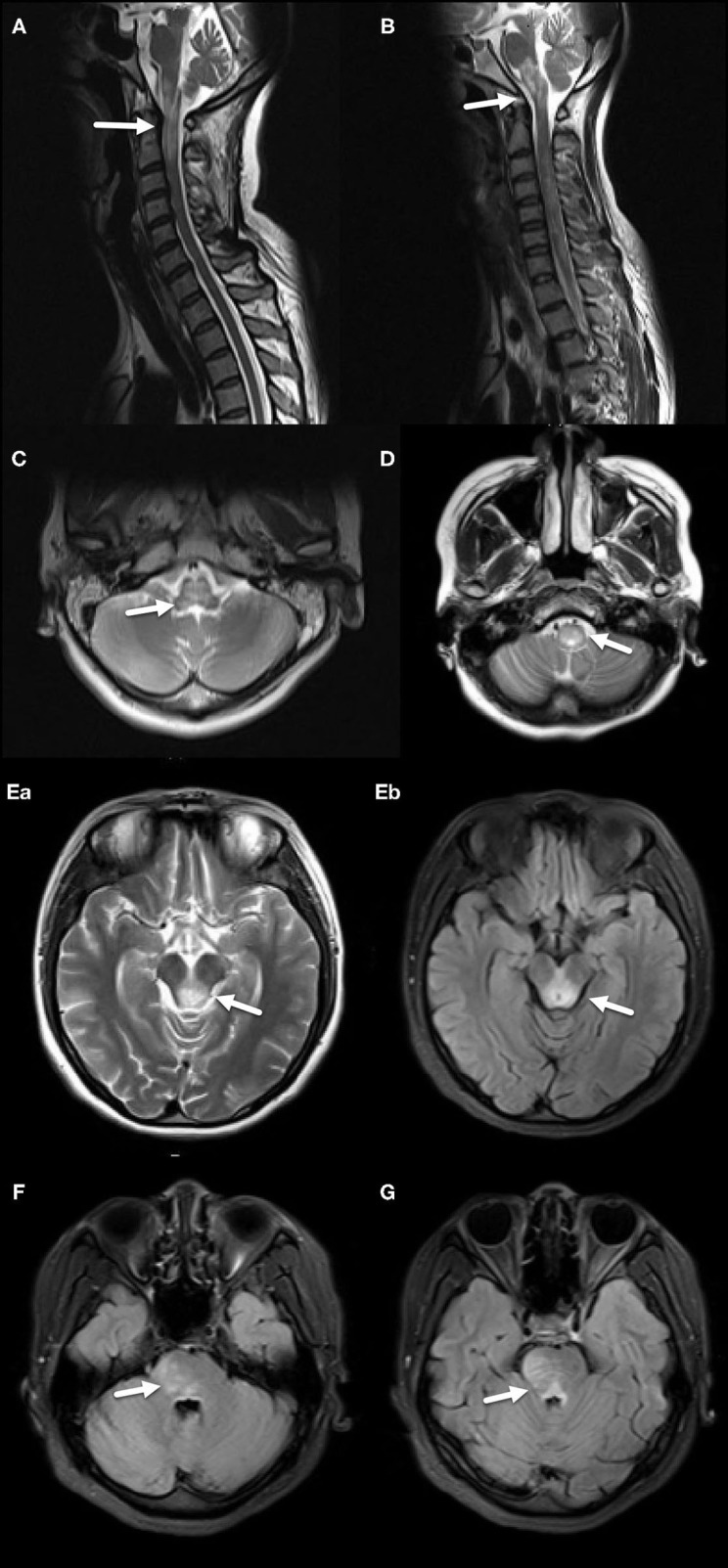
MRI features of the NMOSD group. **(A)** Diffuse, large abnormal signal extending from the junction of cervical spinal cord and medulla oblongata to the upper cervical spinal cord, including AP, indicated by an arrow. **(B)** Extensive diffuse, large abnormal signal in medulla oblongata and AP, indicated by an arrow. **(C)** Large abnormal signal located in central medulla oblongata, indicated by an arrow. **(D)** Large abnormal signal located in central lower medulla oblongata, indicated by an arrow. (**E**–a,b) Large abnormal signal around midbrain aqueduct, indicated by arrows. **(F)** Large abnormal signal located in pons, indicated by an arrow. **(G)** Large abnormal signal located in pons and around the fourth ventricle, indicated by an arrow.

Patients in both groups underwent MRI imaging. In the MS group, there were 26 cases (44.83%) of midbrain lesions, 42 cases (72.41%) of pontine lesions, and 22 cases (37.93%) of medulla oblongata lesions; the NMOSD group had 8 cases (15.38%) of midbrain lesions, 18 cases (34.62%) of pontine lesions, and 46 cases (88.46%) of medulla oblongata lesions. The differences between the groups in all three brain regions were significant (*p* = 0.001, *p* <0.001, *p* <0.001). The number of MS patients with peduncle cerebri and brachium pontis (Middle cerebellar peduncle) lesions was significantly higher than the NMOSD group (*p* = 0.013, *p* = 0.003), while the reverse was true regarding lesions in cervical cord medulla junction and area postrema (AP) (*p* <0.001). MS lesions were peripherally located while the NMOSD lesions were centrally located, both of which were statistically significant (*p* <0.001, *p* <0.001). Moreover, the lesions in the MS group were mostly patchy, while those in the NMOSD group were mostly large (*p* <0.001, *p* <0.001). Importantly, four patients in the MS group showed enhancements while and none in the NMOSD group did, although this data did not achieve statistical significance (*p* = 0.055) (Table 3, Figures 1, 2).

**Table 3:** MRI features of patients.

**Projects**	**MS** **(*n* = 58)**	**NMOSD** **(*n* = 52)**	***P***
Number of lesions = 1 (%)	18 (31.03)	25 (49.00)	0.055
Midbrain lesions	26 (44.83)	8 (15.38)	0.001*
Peduncle cerebri	18 (31.03)	6 (11.54)	0.013*
Tegmental part of midbrain	8 (13.79)	2 (3.85)	0.139
Periaqueduct of midbrain	2 (3.45)	2 (3.85)	1.000
Pontine	42 (72.41)	18 (34.62)	<0.001*
Basal pontine	18 (31.03)	10 (19.23)	0.156
Tegmentum pontine	4 (6.90)	2 (3.85)	0.777
Medulla oblongata	22 (37.93)	46 (88.46)	<0.001*
Dorsal medulla oblongata	6 (10.34)	6 (11.54)	0.841
Ventral medulla oblongata	14 (24.14)	8 (15.38)	0.252
Cervical cord medulla junctional area	2 (3.45)	36 (69.23)	<0.001*
Area postrema (AP)	0	38 (73.08)	<0.001*
Superior cerebellar peduncle	0	1 (1.9)	0.956
Middle cerebellar peduncle (Brachium pontis)	24 (41.38)	8 (15.38)	0.003*
Inferior cerebellar peduncle	0	0	
Peripheral lesions	58 (100)	6 (11.54)	<0.001*
Central lesions	0	46 (88.46)	<0.001*
Lesion morphology			
Patchy lesions	50 (86.21)	12 (23.07)	<0.001*
Large lesions	4 (6.90)	40 (76.92)	<0.001*
Ovoid	5 (8.6)	0	0.088
Linear	0	0	
Enhancing lesions	4 (6.90)	0	0.055

* *P <0.05.*

*Large lesions: diameter is >2 cm (13, 14), or the lesion is larger than 1/4 of the MRI brainstem or spinal cord cross section*.

### Immunological Manifestations

CSF testing was performed in all patients. CSF from the NMOSD group had more WBCs than the MS group (*P* = 0.017). In the MS group, 58 patients were tested for oligoclonal bands (OB) + 24-h IgG synthesis rates, among which 50 (86.21%) were positive for the specific IgG oligoclonal band (SOB). A total of 48 patients with MS were tested for aquaporin-4 (AQP4) Abs in serum and cerebrospinal fluid, and all of them were negative. 16 MS patients were tested for Myelin basic protein (MBP), of which six were positive (37.5%). 52 patients in the NMOSD group were tested for OB + 24-h IgG synthesis rate, of which 8 (15.38%) were positive for the CSF-specific IgG oligoclonal band (SOB). The serums of 46 patients with NMOSD were tested for AQP4 Ab, of which 42 cases were positive (91.30%). Of the 46 patients with NMOSD who had their serums tested for AQP4 Ab, 36 cases were tested for AQP4 Ab in CSF and it was found that 20 cases were positive (55.56%), including one patient whose serum AQP4 Ab was negative. MBP was measured in 16 patients with NMOSD, which revealed eight (50%) positive cases (Table 4).

**Table 4:** Immunological index.

**Immunological index, *n* (positive/total, %)**	**MS** **(*n* = 58)**	**NMOSD** **(*n* = 52)**	***P***
White blood cell (10^6^/L)	7.05 ± 6.04	18.34 ± 19.1	0.017*
Total protein (mg/dl)	28.68 ± 16.28	36.93 ± 21.51	0.160
SOB	50 (50/58, 86.21)	8 (8/52, 15.38)	<0.001*
Median 24 h IgG synthesis rate (mg/24 h)	9.20 (1.06–31.32)	8.49 (1.67–30.75)	0.388
AQP4-Ab	0 (0/48, 0)	42 (42/46, 91.30)	<0.001*
MBP	6 (6/16, 37.50)	8 (8/16, 50.00)	1.000

* *P <0.05.*

*MBP > 0.55 nmol/L is positive. The normal value of 24 h IgG synthesis rate is 0–9mg/24 h*.

## Discussion

MS and NMOSD have been regarded as two distinct diseases, with distinct pathophysiology and clinical courses. The mechanism of MS is multifaceted, though it is generally regarded as an autoimmune disease induced by T cells. However, it has been revealed recently that B cells may play a greater role in MS, complicating the pathology (15, 16). The classic hallmark of MS is multifocal lesions in the central nervous system which vary in location and onset, exhibiting periods of remission. These lesions are characterized by demyelination, relative preservation of axons, inflammatory reactions, glial hyperplasia, and myelin regeneration. The clinical course of MS is diverse, and its lesions can accumulate in most parts of the central nervous system including white matter, optic nerve, brainstem, cerebellum, and spinal cord. On the other hand, NMOSD is a rare disease mediated by antibodies in the central nervous system and is related to serum aquaporin-4 immunoglobulin G antibody (AQP4-IgG). Contrary to MS, the pathophysiology of NMOSD is mediated by primary injury of astrocytes, while demyelination is a secondary manifestation of the disease (1, 17). Furthermore, NMOSD tends to show tropism in the optic nerve, AP, spinal cord, brainstem, diencephalon, and posterior part of the brain (1).

The present study found that both MS and NMOSD were more common in females compared to males, consistent with previous reports (17–21). However, we also found that NMOSD had a greater skew in the ratio of females to males. Additionally, NMOSD had a stronger association with autoimmune diseases and/or autoimmune antibodies (22–24) and involved the elderly population. Our findings concur with prior research which report that the average age of NMOSD patients in the world is between 32.6 and 47.5 years old, with patients over 50 years old accounting for about 25% of the incidence (19, 25, 26).

Previous studies have suggested that NMOSD patients have more serious dysfunction than patients with MS (14, 27), although our data did not find statistical difference in EDSS scores between the two groups at the first onset. This may be due to the fact that our EDSS scores were collected based on patient presentations at symptom onset as compared to the peak of their symptoms. Clinically, the present study found that MS had significantly greater association with MLF and ophthalmoplegia as compared to NMOSD, while NMOSD had greater association with central hiccups, central vomiting, and pyramidal tract signs. Although the MS group had headaches (4/58), paralysis (2/58), and trigeminal neuralgia (1/58) with none in the NMOSD, the difference did not reach statistical significance.

On MRI, MS had significantly more lesions in midbrain and pons compared to the NMOSD group, especially in peduncle cerebri. On the other hand, NMOSD had significantly more lesions in medulla oblongata, especially in the cervical cord medulla junction and AP. Although the MS group had exclusively peripheral lesions (58/58), the NMOSD group's lesions were mostly centrally located. These were consistent with the established knowledge that NMOSD lesions are classically located around ependyma. While the MS lesions were patchy and smaller, NMOSD lesions were large with blurry boundaries. 5 of 58 patients in the MS group had ovoid lesions with clearly defined borders. In the NMOSD group, there was no ovoid lesions with clear boundaries or any linear lesions, although previous reports showed that NMOSD patients had linear lesions (1, 13). There were also no linear lesions in the MS group.

Given that MS and NMOSD have distinct etiologies, pathogeneses, and prognoses, it is unsurprising that their current treatments differ. The main goal of MS therapy is to reduce the chance of recurrence and formation of new lesions, minimizing morbidity and disability (5, 6). On the other hand, NMOSD therapeutic guidelines aim to accelerate the recovery of acute exacerbations, prevent long-term relapse, and minimize chronic sequelae (4). Although the treatment plans of both aim to reduce disease recurrences, NMOSD therapy is generally comprised of immunosuppressive drugs, unlike the immunomodulators that are commonly used in MS (7). Some disease modifying drugs (DMDs) for MS, such as interferon-β, fingolimod, and natalizumab, have been found to be not only ineffective but actually aggravate the condition of NMOSD patients (4, 8, 9). Therefore, it is crucial to correctly diagnose these patients as soon as possible and provide the correct treatment.

Clinically, it is observed that some MS and NMOSD patients have partially overlapping symptoms when they initially present with brainstem lesions. Unsurprisingly, this makes diagnosis difficult, delaying early immunological intervention treatment and, therefore, patient prognosis. Thus, the present study sought to clarify diagnostic decision-making by characterizing the clinical manifestations and imaging features in patients who initially present with symptoms of brainstem involvement.

The differences in clinical and radiographical presentations described by this study may assist in differentiating between MS and NMOSD in clinical practice. Furthermore, much of our findings align with prior research. In terms of symptomatic presentations, previous research suggested that ophthalmoplegia is the most common abnormality in MS patients with brainstem lesions, specifically in the medial longitudinal fasciculus (28–30), while central hiccups and central vomiting are pathognomonic for NMOSD patients (31–33). These findings were consistent with the present study. Meanwhile, previous studies did not note that NMOSD patients had pyramidal symptoms. Our study found that both MS and NMOSD groups experienced pyramidal symptoms, with significantly greater association with NMOSD. On the other hand, previous studies had reported cases of pupillary miosis in patients with MS and NMOSD (34), but this phenomenon was not observed in the present study.

On MRI imaging, Jurynczyk et al. found that MS was distinct from myelin oligodendrocyte glycoprotein (MOG)-Antibody associated disease (MOGAD) and NMOSD, while it was difficult to distinguish MOGAD from NMOSD by imaging alone due to overlapping features. They further described that MS presented peripherally with ovoid lesions adjacent to the body of lateral ventricles (e.g., Dawson's fingers) or within the U fibers as T1 hypointense lesions. Meanwhile, they described MOGAD and NMOSD lesions as large, poorly demarcated (fluffy) brainstem lesions centrally located in pons and/or adjacent to the fourth ventricle, at the periaqueductal region or in area postrema. Overall, MOGAD/NMOSD had fewer lesions as compared to MS (13, 35). These aligned with our study's findings, which found that NMOSD has centrally located larger lesions in fewer quantities.

Similarly, Nakamura's group described that although both MS and NMO present with lesions in the corpus callosum, the qualities are distinctive. MS presented as small lesions at the corpus callosum, both acutely and chronically. Meanwhile, NMO only presented acutely as large, edematous legions with heterogeneous intensity, also described as a marble pattern. NMO lesions disappeared at the chronic stage (36). Meanwhile, in the spinal cord, MS presented peripherally in the lateral and posterior white matter regions, while NMO presented in the central gray matter; in the acute stage, NMO lesions covered more than half the cord (37). These descriptions aligned with our findings that MS tended to present as small, numerous lesions in the periphery, while NMO tended to present as large, fewer lesions in the central regions.

Matsumoto's group was able to tease apart MOGAD related imaging findings from NMOSD findings. They found that MOGAD had more subcortical white matter lesions of temporal lobe and cerebellar peduncle, and pyramidal and medial medulla lesions, while NMOSD had more lesions in dorsal medulla and area postrema. These aligned with our findings that NMOSD presented with lesions commonly in the medulla oblongata, although our data did not suggest that it was localized to a specific region as Matsumoto's group described (38).

Hayashida's group further elaborated on NMOSD's presentation in the spinal cord. They described that more than half of NMOSD lesions occupied the posterior and lateral columns, while only roughly a third was located in the anterior column, posterior horn, central portion, and anterior horn (39). Although these data agree with our findings that NMOSD lesions occupy gray matter, our study did not find the lesions to preferentially target white matter in the spinal cord columns.

In conclusion, it was found that most MS and NMOSD patients initially presenting with brainstem involvement are female, a fact which is magnified in NMOSD patients. NMOSD patients were more likely to be accompanied by autoimmune diseases or positive autoimmune antibody testing, and were more likely to exhibit central hiccups, central vomiting, and pyramidal tract signs. Lesions in NMOSD patients were mostly found in medulla oblongata, in the shape of large sheets, and were centrally located. Conversely, MS patients were more likely to experience ophthalmoplegia and MLF syndrome, and exhibit patchy, peripheral lesions mostly appearing on the midbrain and pons. Ultimately, these features may offer the means to create an algorithm that clinicians can follow in order to diagnose MS and NMOSD patients who present with brainstem lesions as the first manifestation, ultimately expediting initiation of treatment to improve overall prognosis.

## Data Availability Statement

The original contributions presented in the study are included in the article/supplementary material. Further inquiries can be directed to the corresponding author(s).

## Ethics Statement

Ethical review and approval was not required for the study on human participants in accordance with the local legislation and institutional requirements. Written informed consent for participation was not required for this study in accordance with the national legislation and the institutional requirements. Written informed consent was not obtained from the individual(s) for the publication of any potentially identifiable images or data included in this article.

## Author Contributions

QJ, ZL, and YT prepared the manuscript. HD, YD, and XG designed the study. YD, KE, YH, and HL revised the manuscript. All authors reviewed the manuscript and provided the final approval for the manuscript.

## Conflict of Interest

The authors declare that the research was conducted in the absence of any commercial or financial relationships that could be construed as a potential conflict of interest.

## Publisher's Note

All claims expressed in this article are solely those of the authors and do not necessarily represent those of their affiliated organizations, or those of the publisher, the editors and the reviewers. Any product that may be evaluated in this article, or claim that may be made by its manufacturer, is not guaranteed or endorsed by the publisher.
